# Effect of green Spanish‐style Manzanilla packaging conditions on the prevalence of the putative probiotic bacteria *Lactobacillus pentosus* TOMC‐LAB2

**DOI:** 10.1002/fsn3.272

**Published:** 2015-09-23

**Authors:** Francisco Rodríguez‐Gómez, Verónica Romero‐Gil, Pedro García‐García, Francisco Noé Arroyo‐López, Antonio Garrido‐Fernández

**Affiliations:** ^1^ Food Biotechnology DepartmentInstituto de la Grasa (Agencia Estatal Consejo Superior de Investigaciones Científicas, CSIC) Campus Pablo de Olavide, Building 46. Ctra. Utrera, km 1.41013SevilleSpain

**Keywords:** Lactic acid bacteria, packaging conditions, probiotic, table olives, yeasts

## Abstract

This work focuses on the persistence of the putative probiotic bacteria *Lactobacillus pentosus* TOMC‐LAB2 on green Spanish‐style Manzanilla olives according to different packaging conditions and storage temperatures. The lactic acid bacteria population decreased with time but the highest survival counts (and lowest yeasts) at the end of storage (8 months) were observed in plastic pouches under nitrogen atmosphere and glass jars with brine stored at 20°C. Molecular techniques showed a 100% presence of the putative probiotic bacteria in biofilms adhered to olive epidermis, while it was absent in PPB (plastic pouches with brine) and in olives stored at 7°C. No changes in NaCl, pH or combined acidity were observed during the storage except for a slight increase in titratable acidity at 20°C. The color of the fruits was stable but degraded at 20°C for olives in plastic pouches with brine.

## Introduction

The production of green Spanish‐style table olive is a traditional process which is usually achieved by spontaneous fermentation at ambient temperature. The main group of microorganism involved in the fermentation process is LAB (lactic acid bacteria), but yeasts are always present throughout processing (Garrido‐Fernández et al. 1997; Arroyo‐López et al. 2012a). In general, the genus *Lactobacillus* is recognized as the most characteristic of the indigenous LAB involved in green Spanish‐style fermentation (Hurtado et al. 2012; Doulgeraki et al. 2013). On the contrary, the most frequent yeast genera isolated from table olive processing have been *Candida, Pichia, Debaryomyces*, and *Saccharomyces* (Arroyo‐López et al. 2012a; Botta and Cocolin 2012).

Table olives were a convenient carrier for delivering probiotic bacteria to humans (Lavermicocca et al. 2005) and their abilities in this aspect are comprehensively discussed in a recent review (Peres et al. 2012). Initial studies were based on the adhesion and survival of nonnative lactobacilli and bifidobacteria species. In this way, *Lactobacillus paracasei* IMPC2.1 (isolated from humans) had a lengthy survival on olives and could be recovered from the fecal samples of consumers (Lavermicocca et al. 2005). This organism survived in olive fermentations but olives showed a considerably genetic diversity of LAB species (De Bellis et al. 2010). However, autochthonous microorganisms may also colonize the olives during fermentation and, in principle, due to their origin, have a major survival and imposition (Nychas et al. 2002). The formation of bacteria‐yeast communities (true biofilms) on Gordal olives during fermentation has been described (Domínguez‐Manzano et al. 2012). Several LAB strains selected by their potential probiotic characteristics not only fermented the olives but formed mixed biofilms (composed of LAB and yeasts) on Manzanilla olives (Arroyo‐López et al. 2012b; Bautista‐Gallego et al. 2013). Further studies reported that inoculation assured a favorable competition with the wild strains but did not guarantee *per se* the complete imposition (Rodríguez‐Gómez et al. 2013). Blana et al. (2014) has reported high colonization (80–90%) of *Lactobacillus pentosus* B281 and B282 at 8% NaCl but the second was unable to establish on the olive surface. In heat‐shocked olives, the former exhibited higher percentages of recovery (Argyri et al. 2014). Other examples on the use of starter cultures for the production of probiotic vegetables are sauerkraut, Irish brassica, or cereal‐based substrates (Charalampopoulus et al. 2003; Verganović et al. 2011; Jaiswal et al. 2012).

But not only the survival and imposition of the probiotic LAB in the polymicrobial biofilms at the end of the fermentative process is important, but also its presence at the end of the storage and packaging. The effect of packaging material, storage conditions, or the food/LAB relationships may affect the functional efficacy (Ranadhera et al. 2010; Rodríguez‐Gómez et al. 2015). Natural black Conservolea olives packaged in polyethylene pouches showed a high heterogeneity among LAB but the species were, as usual, *L. pentosus* and *Lactobacillus platarum*. The composition of the packaging atmosphere (aerobic or modified) influenced the yeast activity of natural black Conservolea olives packaged in polyethylene pouches (Doulgeraki et al. 2012). The putative probiotic *L. pentosus* TOMC‐LAB2 strain showed a high survival for a long period of time (>6 months) in fortified green Spanish‐style olives (Rodríguez‐Gómez et al. 2014a). However, scarce work has been carried out with the objective of linking the olive biofilm formed during fermentation to the microbial population at the end of larger periods of the packaged products, although this information is essential for producing probiotic olives by controlled fermentation which guarantees, after the adequate packaging conditions, the delivering of the potential functional LAB to consumers.

Therefore, the objective of the present work was to study the changes in the polymicrobial biofilms formed during fermentation (and particularly the presence of the putative probiotic bacteria *L. pentosus* TOMC‐LAB2, henceforth LAB2), throughout a lengthy storage of packaged green Spanish‐style Manzanilla table olives, according to different packaging conditions and storage temperature. The level of imposition of the bacterium at the end of this period was also analyzed. In addition, the evolution during storage of the physicochemical characteristics of brines and the olive color of the previously LAB2‐fermented products were also monitored. The results can contribute to improving information about the changes that the microbial population adhered to olives during fermentation may suffer from packaging until the product reaches the consumer. Eventually, they may also help to redesign current packaging practices or to develop new specific technologies to produce probiotic olives.

## Materials and Methods

### Selected olives

The olives used in this study were from the Manzanilla cultivar, fermented as Spanish‐style during the 2010/2011 season using LAB2 as starter culture. The characteristics of the fermentations have been described elsewhere (Rodríguez‐Gómez et al. 2014b). After fermentation, fruits were manually selected to separate defective olives, washed with sterile water to remove the microbial load not integrated into the olive biofilm, and finally packaged according to the conditions described below.

### Packaging conditions

Packaging was achieved in GJB (glass jars with brine), plastic pouches (composed of a high barrier polyester and polyethylene with a thickness of 109 *μ*m and oxygen permeability of less than 8.5 cm^3^/m^2^/24 h, SP Group, Villarrubia, Spain) with brine (PPB), or plastic pouches under N_2_ atmosphere without brine (PPN). These packaging conditions were selected due to their promising behavior in previous studies on overall LAB survival during packaging (Rodríguez‐Gómez et al. 2015); however, a longer study and the presence of the inoculated strain in the final product require further confirmation. The GJB containers were filled with 125 g of olives and 125 mL of sterile brine. The PPB pouches were filled with 75 g olives which were covered with 75 mL of sterile brine. The PPN pouches were filled with 75 g olives and 30% of its displaced free volume replaced with N_2_. Plastic pouches were closed manually using a Tecnotrip mod EVT7G (Terrassa, Spain) closing machine.

In this work, the physicochemical conditions developed by Borbolla y Alcalá and González Pellissó (1972) for long‐term green Spanish‐style table olive preservation and the limits established in the Trade Standard Applying to Table Olives (IOC, 2004) for packaging of this product were used. Accordingly, the equilibrium concentrations were fixed at 5 g NaCl 100 mL^−1^ and 0.5 g lactic acid 100 mL^−1^. The use of the minimum allowed salt level is compulsory due to the LAB sensitivity to salt (Romero‐Gil et al. 2013). Before packaging, the cover brines were sterilized (121°C, 15 psi for 15 min) to prevent any contamination of the packaged olive microbiota. Olives packaged under nitrogen atmosphere were previously equilibrated at the above‐mentioned conditions in sterilized glass containers stored for 20 days in a cold‐storage room at 7°C. Packaged olives from all treatments (GJB, PPB, and PPN) were divided into two sublots which were stored at 7 ± 1°C (to mimic conditions prevailing during a possible distribution of the product throughout a cold chain) and 20 ± 2°C (for mimicking distribution at environmental temperature). Therefore, the experiment consisted of a full factorial design with a total of six treatments (3 packaging methods × 2 storage temperatures). Duplicated samples from each treatment were periodically withdrawn (without replacement) during 8 months.

### Microbiological analysis

Brine samples were diluted, if necessary, in a sterile saline solution (0.9 g 100 mL^−1^ NaCl) and plated using a Spiral System model dwScientific (Dow Whitley Scientific Limited, Shipley, West Yorkshire, England) on appropriate media. *Enterobacteriaceae* were counted on Crystal‐violet Neutral‐Red bile glucose agar (Merck, Darmstadt, Germany), LAB were spread onto de Man‐Rogosa and Sharpe agar (Oxoid, Cambridge, UK) supplemented with 0.02% (wt/vol) sodium azide (Sigma, St. Luis, MO), and yeasts were grown on a YM (yeast–malt–peptone–glucose medium) agar (Difco, Becton and Dickinson Company, Sparks, MD) supplemented with oxytetracycline and gentamicin sulfate (0.005%, wt/vol) as selective agents for yeasts. The plates were incubated at 30°C for 24 (*Enterobacteriaceae)* or 72 h (yeasts and LAB) and counted using a CounterMat v.3.10 (IUL, Barcelona, Spain) image analysis system. Brine counts were expressed as log_10_ CFU mL^−1^.

The microbial population dynamics on olives was followed by determining the microorganisms adhered to the olive epidermis, using the enzymatic protocol developed by Böckelmann et al. (2003) for the detachment of biofilms. Briefly, three fruits from each packaging vessel were randomly taken and washed for 1 h with 250 mL of a sterile PBS buffer solution. Then, the olives were transferred to 50 mL of a PBS solution with the following enzymes: 14.8 mg L^−1^ lipase (L3126), 12.8 mg L^−1^ *β*‐galactosidase (G‐5160), and 21 *μ*L L^−1^ *α*‐glucosidase (G‐0660) (Sigma‐Aldrich, St. Louis, MO). To achieve biofilm disintegration and removal of the adhered cells, the fruits were incubated at 30°C in this enzyme cocktail with slight shaking (150 rpm). After 12 h, the olives were removed and the resulting suspension was centrifuged at 9000 × *g* for 10 min at 4°C. Finally, the pellet was resuspended in 2 mL of PBS and spread onto the different culture media described above. Olive counts were expressed as log_10_ CFU olive^−1^. Expression in other units can easily be achieved considering an average (obtained from 50 fruits) weight of 4.08 ± 0.46 g and an olive surface of 10.99 ± 1.01 cm^2^ per fruit.

The analyses were performed, in duplicate (two packages) and simultaneously, for brines and olives from each treatments at 0, 60, 120, 180, and 240 days (8 months).

### Characterization of the lactic acid bacteria population

For characterization of the lactobacilli population, repetitive bacterial DNA element fingerprinting analysis (rep‐PCR) with primer GTG_5_ was followed (Gevers et al. 2001). The PCR reaction in a final volume of 25 *μ*L contained: 5 *μ*L of 5× MyTaq reaction buffer (5 mmol L^−1^ dNTPs and 15 mmol L^−1^ MgCl_2_), 0.1 *μ*L of My Taq DNA polymerase (Bioline reactives, London, United Kingdom), 1 *μ*L GTG_5_ primer (25 *μ*m), 13.9 *μ*L deionized H_2_0, and 5 *μ*L DNA (~20 ng *μ*L^−1^). PCR amplification was carried out in a thermal cycler (Master Cycler Pro; Eppendorf, Hamburg, Germany) with the following program: 95°C for 5 min as initial step, plus 30 cycles at: (1) 95°C for 30 sec; (2) annealing at 40°C for 30 sec; and (3) 65°C for 8 min, with a final step of 65°C for 16 min to conclude the amplification. This methodology was used to determine the recovery frequency of the LAB2 strain at the end of packaging. Firstly, the repeatability of the technique was determined using the same LAB2 strain as the internal control, obtaining 86.9 ± 3.4% of reproducibility (data not shown). Then, diverse isolates (a total of 20) were randomly picked from each treatment at the end of the experiment (8 months), making a total of 120 lactobacilli to analyze (60 isolates at 7°C and 60 isolates at 20°C). They were named according to packaging treatment (GJB, PPB, or PPN). Their pattern profiles of bands (from 100 up to 3000 bp) were compared with the strain used to inoculate initially the fermentation process (LAB2). For this purpose, the PCR products were electrophoresed in a 2% (wt/vol) agarose gel and visualized under ultraviolet light by staining with ethidium bromide. The resulting fingerprints were digitally captured and analyzed with the BioNumerics 6.6 software package (Applied Maths, Kortrijk, Belgium). The similarity among digitalized profiles was calculated using the Pearson product‐moment correlation coefficient. Dendrograms were obtained by means of the UPGMA (Unweighted Pair Group Method using the Arithmetic Average) clustering algorithm and the automatic calibration tool for the determination of the optimization and curve smoothing parameters.

### Physicochemical analyses

The analyses of cover brines for pH, salt concentration (g NaCl 100 mL^−1^ brine), titratable (expressed as g lactic acid 100 mL^−1^ brine), and combined acidities (expressed as milliequivalents of HCl added to 1 L brine to reach pH 2.6) were carried out using the standard methods developed for table olives (Garrido‐Fernández et al. 1997). Each parameter was measured in duplicate and the average recorded.

The firmness of olives was measured at the beginning and at the end of the storage using a Kramer shear compression cell coupled to an Instron Universal Machine (Canton, MA). The crosshead speed was 200 mm min^−1^. The firmness, expressed as N 100 g^−1^ flesh, was the mean of ten replicate measurements, each of which was performed on three pitted olives.

The instrumental surface color of the fruits was measured using a BYK‐Gadner Model 9000 Colour View Spectrophotometer (Silver Spring, MD). Any interference from stray light was minimized by covering the samples with a box, which had a matt black interior. Color was expressed as *CI* (Colour Index) calculated according to (Sánchez‐Gómez et al. 1985):

$$\mathrm{CI}=\frac{-2*R_{560}+R_{590}+4*R_{635}}{3},$$

where *R* stands for the reflectance at 560, 590, and 635 nm, respectively. Olive color was also determined in terms of the CIE L*, a*, b* parameters. Results were the average of 10 measurements.

### Statistical data analysis

Comparisons among variable levels were obtained after General Linear model (GLM) analysis by Least Significant Difference. In the graphs, average means with nonoverlapping confident limits are significantly different at a probability level of *P* < 0.05. Data analysis was carried out using Statistica 8.0 software package (StatSoft Inc, Tulsa, OK).

## Results

### Microbial population changes

**Figure 1:**
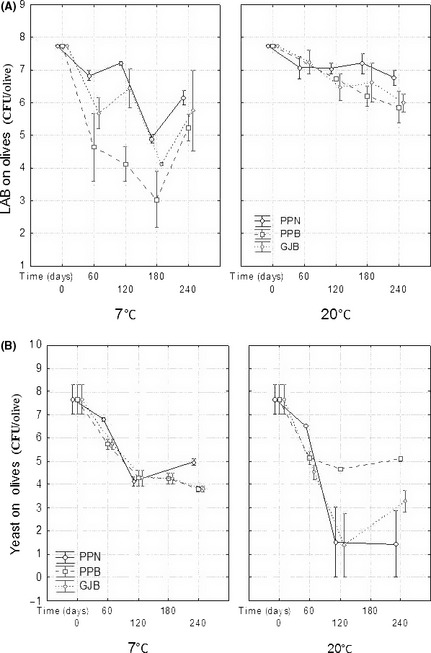
Lactic acid bacteria (panel A) and yeast (panel B) average (*n* = 2) changes on olive biofilm as a function of time, according to packaging conditions. GJB, packaging in glass jars with brine; PPB, packaging in plastic pouches with brine; PPN, packaging in plastic pouches under nitrogen atmosphere; weighted means; vertical bars denote ± standard errors.

**Figure 2:**
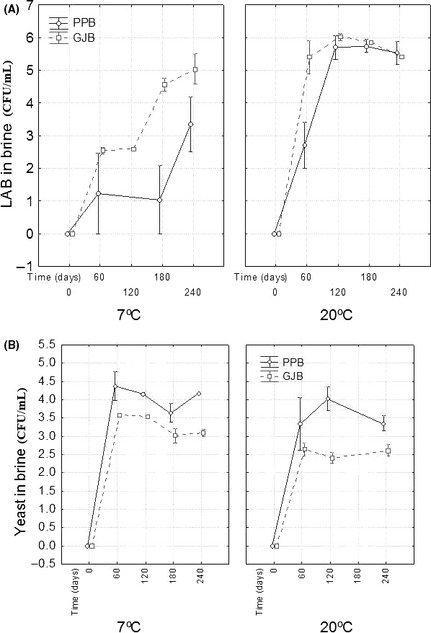
Lactic acid bacteria (panel A) and yeast (panel B) average (*n* = 2) changes in brines with time, according to packaging conditions. GJB, glass jars with brine; PPB, plastic pouches with brine; weighted means; vertical bars denote ± standard errors.

The evolution of the microbial load on olives as a function of time was studied for the three packaging conditions assayed while the changes in brines were monitored only for the GJB and PPB treatments (Figs. 1, 2). On olives, there was a general decrease in the LAB and yeast populations with significant effects due to temperature and packaging conditions on specific sampling points (Fig. 1). The greatest survival of LAB populations on olives was achieved at 20°C with only limited (although significant in some cases) differences among packaging conditions (Fig. 1A, right). Interestingly, at this temperature, PPN had the highest significant population levels at the end of the study (Fig. 1A, right). Overall, changes in LAB population moved from 7.77 ± 0.02 log_10_ CFU olive^−1^ (at the onset of the experiment) to 5.86 ± 0.50 (PPB) or 6.78 ± 0.23 log_10 _CFU olive^−1^ (PPN) (after 8 month storage). Hence, storage at 20°C led to a fairly good LAB population survival on olives, particularly in PPN packaging where the counts only decreased 1 log cycle after an 8‐month storage (a lengthy period for probiotic olives, and tested in this work for the first time). On the contrary, the decrease was higher at 7°C (Fig. 1A, left), particularly in PPB which showed markedly significant and the lowest averages during the central period of the storage. At this temperature, the olives packaged in PPN also had the best behavior and its curve was, in general, above those of the other packaging conditions (Fig. 1A, left). Therefore, in general, the greatest LAB survival was observed when olives were stored at 20°C, particularly in PPN packaging (only 1 log_10_ cycle decrease), followed by GJB and PPB (2 log_10_ cycle decrease).

There was also a marked decrease in the yeast populations on olives (Fig. 1B). At 20°C, the decrease observed in olives packaged in PPN or GJB was very marked and sharp, while in PPB the reduction was moderate and similar to that shown at 7°C (Fig. 1B, right). At 7°C, the decrease was noticeably significant, regardless of packaging methods, but the population stabilized during the last storage period at levels between 5.0 (PPN) and 4.0 log_10 _CFU olive^−1^ (GJB and PPB) (Fig. 1B, left). Apparently, there was an apparent inverse relationship between LAB and yeast survival at 20°C which depended on the packaging method. However, the same effect was hardly appreciable at 7°C (Fig. 1B, left) probably because this temperature was more stressing for LAB than for yeasts. Therefore, LAB population survival was better preserved at 20°C but at the expense of a lower yeast population; on the contrary, LAB survival was lower at 7°C and its decrease propitiated a higher yeast survival in the long run.

In brines, LAB and yeasts were initially below the detection limits due to the initial sterilization of the packaging brine; later, LAB and yeasts from the olive biofilm were released into the brine, and their populations increased progressively with a significant effect due to packaging conditions and temperature (Fig. 2). The LAB increase was faster and reached higher average populations at 20°C than at 7°C, except at the end of the storage; in most of the sampling points GJB brines also showed the greatest average population (Fig. 2A). On the contrary, the yeasts increased faster and reached their highest initial populations at 7°C, particularly in PPB (Fig. 2B). Therefore, the same opposed behavior between LAB and yeast populations described on olives was also observed in brine; a slower increase in LAB and lower populations led to faster increase and higher populations of yeasts. The effect was particularly evident at 7°C where the LAB increase was rather slow (mainly in PPB) and reached lower averages (Fig. 2A, left). At 20°C, the effect of packaging conditions on LAB increase was appreciated only during the first half period of the storage (Fig. 2A, right) but the position of the yeast curves in the same containers (Fig. 2B, right) were also reversed with respect to the position of those of the LAB and showed a marked lower maximum population in GJB than in PPB. Therefore, the effects of the packaging materials on LAB and yeasts in the brine were also opposed, as on counts on olives, with GJB leading to the highest LAB counts and PPB to the highest yeast populations.

### Characterization of the lactobacilli population obtained from olive biofilm at the end of storage

**Figure 3:**
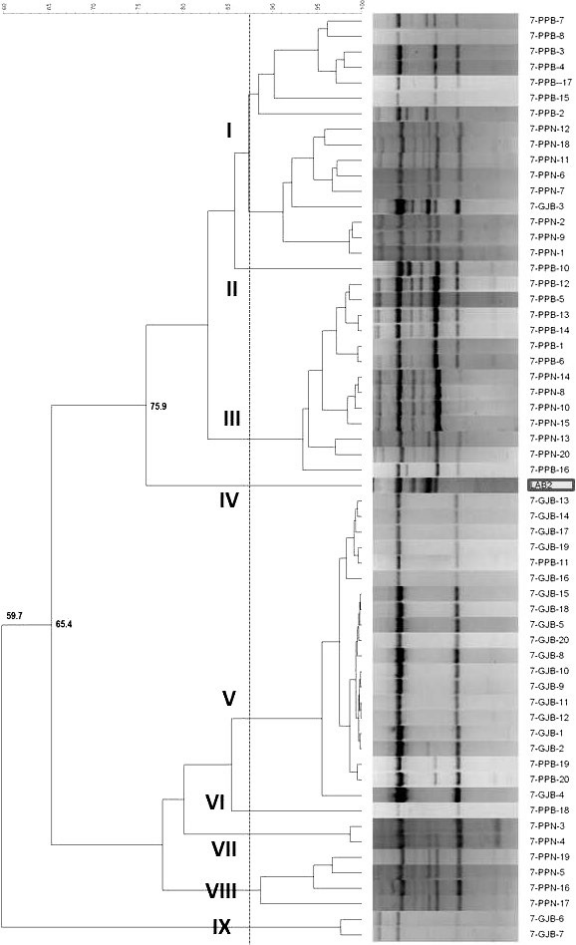
Dendrogram generated after cluster analysis of the digitalized rep‐PCR fingerprints with primer GTG_5_ of LAB isolates obtained from olives stored at 7°C. Clustering parameters: 0.5% optimization and 3.75% curve smoothing. GJB, glass jars with brine; PPB, plastic pouches with brine; PPN, plastic pouches under nitrogen atmosphere.

The presence of the LAB2 strain at the end of packaging storage was determined by molecular methods (rep‐PCR). Figure 3 shows the dendrogram generated using the pattern profiles of the 60 lactobacilli isolates obtained, after 8 months of packaging, from the treatments stored at 7°C. The cluster analysis showed that a total of nine major groups were differentiated below reproducibility of the technique and associated error (86.9%). The LAB2 isolate used as an internal control formed a cluster (IV) clearly separated from the rest of the lactobacilli. This is indicative that the putative probiotic strain LAB2 is not present at the end of the storage in the treatments stored at 7°C. With respect to the (wild) LAB population on olives at a low temperature, a total of eight different wild genotypes were found among LAB isolates, with the cluster V containing 20 isolates (most of them obtained from GJB treatment) as the most important, while treatments in plastic pouches (PPB and PPN) showed a high biodiversity.

**Figure 4:**
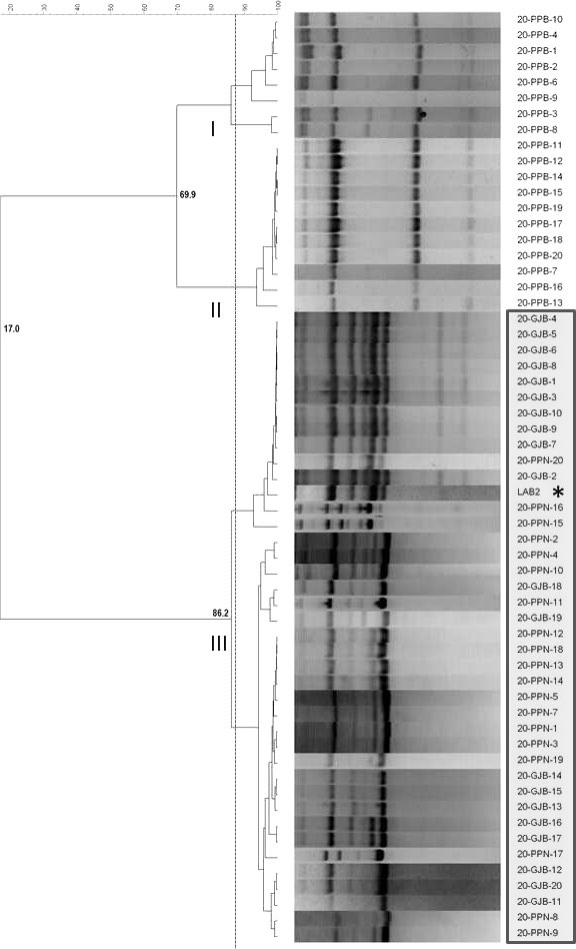
Dendrogram generated after cluster analysis of the digitalized rep‐PCR fingerprints with primer GTG_5_ of lactic acid bacteria isolates obtained from olives stored at 20°C. Clustering parameters: 2.0% optimization and 3.75% curve smoothing. GJB, glass jars with brine; PPB, plastic pouches with brine; PPN, plastic pouches under nitrogen atmosphere.

Figure 4 shows the dendrogram generated using the pattern profile of the 60 lactobacilli isolates obtained at the end of storage from the treatments at 20°C. The cluster analysis showed that a total of three major groups were differentiated below reproducibility of the technique. In this case, cluster III included the LAB2 strain used as internal control as well as another 43 isolates. Thereby, storage at room temperature (20°C) had favored considerably the permanence of the LAB2 strain in selected packaging conditions, mainly in treatments with nitrogen atmosphere (PPN) and GJB, where the recovery frequency of the inoculum was 100%. On the contrary, the inoculum was not detected in PPB (plastic pouches with brine) (recovery frequency 0%) and two wild genotypes (clusters I and II) were found.

### Effect of the packaging treatments on the physicochemical characteristics

**Figure 5:**
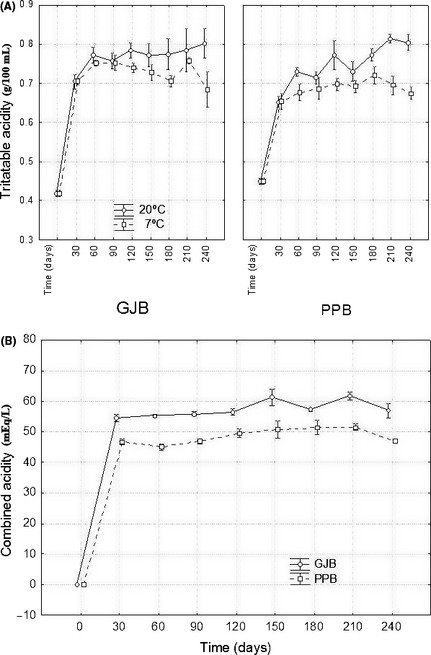
Changes in the averages of titratable (panel A) and combined acidity (panel B) (*n* = 4) during storage (time), according to packaging conditions and storage temperature. GJB, glass jars with brine; PPB, plastic pouches with brine; weighted means; vertical bars denote ± standard errors.

The changes in NaCl, pH, and combined acidity were appreciated only during the first 30 days after packaging; later, due the equilibrium between brine and flesh, the levels remained stable. Final values of the physicochemical characteristics in GJB were: pH 3.68 (average) ± 0.02 (standard error) and 3.74 ± 0.03 for storage at 20 and 7°C, respectively; NaCl, 5.50 ± 0.06 and 5.48 ± 0.07 g 100 mL^−1^; and combined acidity 57 ± 5 and 57 ± 1 mEq L^−1^. In PPB, the levels were: pH, 3.52 ± 0.04 and 3.55 ± 0.02; NaCl, 5.57 ± 0.04 and 5.59 ± 0.02 g 100 mL^−1^; and combined acidity, 48 ± 1 and 46 ± 1 mEq L^−1^, for 20 and 7°C, respectively. Hence, the values in the equilibrium for the main variables responsible for the preservation of this type of olive were appropriate and the packages remained stable during the 8 months of the experiment monitoring. Titratable acidity was always (and sometimes significantly) higher in packages stored at 20°C and showed a first sharp raise, due to its release from the flesh. Then, this parameter showed a steady slight increasing trend at 20°C in packages containing brine, with significant differences between some sampling points within packaging material. This production of lactic acid indicates a certain LAB activity throughout this period. In principle, these changes do not represent any inconvenience and can contribute to improving the stability and safety of the product during commercialization. Finally, although combined acidity did not change during storage, the levels found in GJB were always significantly greater than those in PPB (Fig. 5B), but the difference (only about 10 mEq L^−1^) had a limited influence on the respective storage stabilities.

In the PPN packaging, without brine, the physicochemical characteristics in the flesh after the equilibrium were: NaCl, 5.65 ± 0.03 g 100 g^−1^; pH, 3.51 ± 0.01; titratable acidity, 0.70 ± 0.01 g lactic acid 100 g^−1^; and combined acidity, 54.9 ± 0.5 mEq L^−1^ brine. The periodical analysis of the PPN samples throughout the storage period showed that these parameters remained stable during the entire storage period.

The firmness showed a slight decline from the original value (1377 ± 24 N 100 g^−1^ olive flesh) to final levels (1278 ± 20 N 100 g^−1^ olive fresh in GJB; 1257 ± 26 N 100 g^−1^ olive flesh in PPB olives; and 1385 ± 30 N 100 g^−1^ olive flesh in PPN). There were no significant differences among packaging conditions at any sampling time and with only a slightly but not significant higher average in olives packaged in PPN stored at 7°C. Therefore, from the firmness side, because of the lack of effect of the packaging conditions on this quality attribute during the 8 months of storage, any of them could be used.

Therefore, the physicochemical characteristics prevailing during storage were very close to the values expected and allowed the adequate stability of the products, regardless of packaging materials and storage temperature. The results validate the suitability of the packaging conditions previously fixed (Borbolla y Alcalá and González Pellissó 1972; IOC, 2004) and the calculus habitually used for estimating equilibrium conditions (Garrido‐Fernández et al. 1997).

### Effect of the packaging treatments on the colour parameters

The GLM analysis applied to each parameter and the packaging condition*temperature interaction showed several significant differences at numerous sampling periods. However, this work is mainly interested in investigating general trends. A first, data overview showed that temperature had the lowest influence on color. Hence, a graph of each color parameter versus time, according to packaging conditions, was a convenient option for displaying the interaction; in this case, one must bear in mind that the error includes the variability due to temperature.

**Figure 6:**
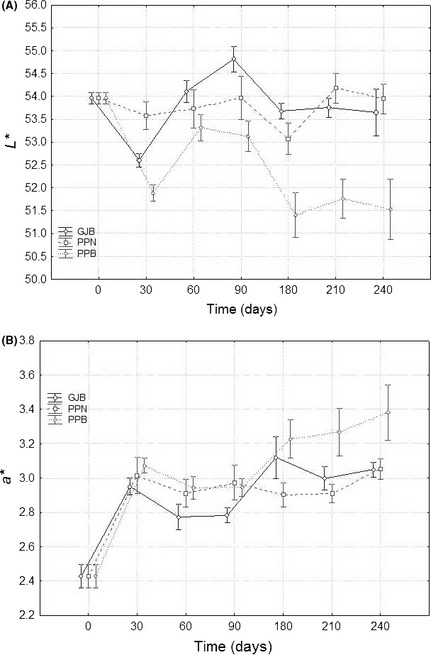
Average (*n* = 8) changes in L* (panel A) and a* (panel B) color parameters during storage (time), according to packaging conditions. Variability due to temperature included in error. GJB, packaging in glass jars with brine; PPB, plastic pouches with brine; PPN, plastic pouches under nitrogen atmosphere; weighted means; vertical bars denote ± standard errors.

**Figure 7:**
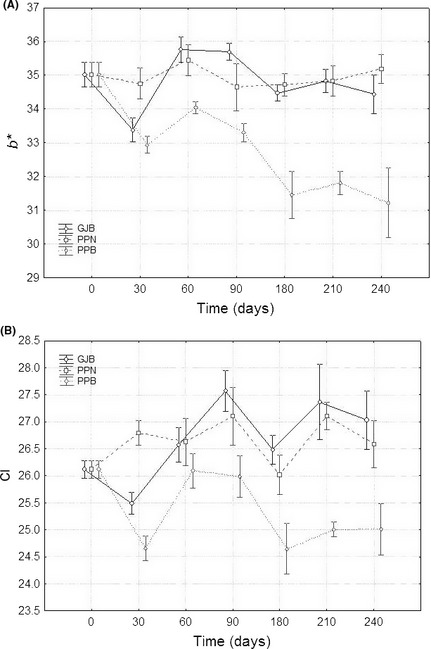
Average (*n* = 8) changes in b* (panel A) and Color Index (panel B) color parameters during storage (time), according to packaging conditions. Variability due to temperature included in error. GJB, glass jars with brine; PPB, plastic pouches with brine; PPN, plastic pouches under nitrogen atmosphere; weighted means; vertical bars denote ± standard errors.

The L* parameter (luminance) (Fig. 6A) was, in general, poorer (lower values) in pouches with brine (PPB) while olives packaged in GJB and PPN followed, in general, a rather similar trend with acceptable color. Therefore, packaging in PPB always led to darker olives. In a* (Fig. 6B), the three packaging conditions followed similar trends during the first 90 days but, later, the values of this parameter increased progressively in the PPB olives which, in the long run, had significant higher values of a* (related to more reddish tones and poorer colors). Similarly, values of b* and *CI* in olives from PPB (Fig. 7) were below those corresponding to GJB and PPN fruits (with similar behavior and better color) throughout the storage. As a result, olives in PPB have faded (b* have moved toward bluer tones) and had lower *CI*; in both cases, these changes are related to color degradation and poorer appearance. Therefore, the values of L*, a*, b*, and *CI* followed a similar trend with time in GJB and PPN olives which maintained their initial color (or even improved it in some cases) for the 8 months that the study lasted. On the contrary, all the color parameters showed poorer values in PPB shortly after packaging and throughout the entire storage period.

**Figure 8:**
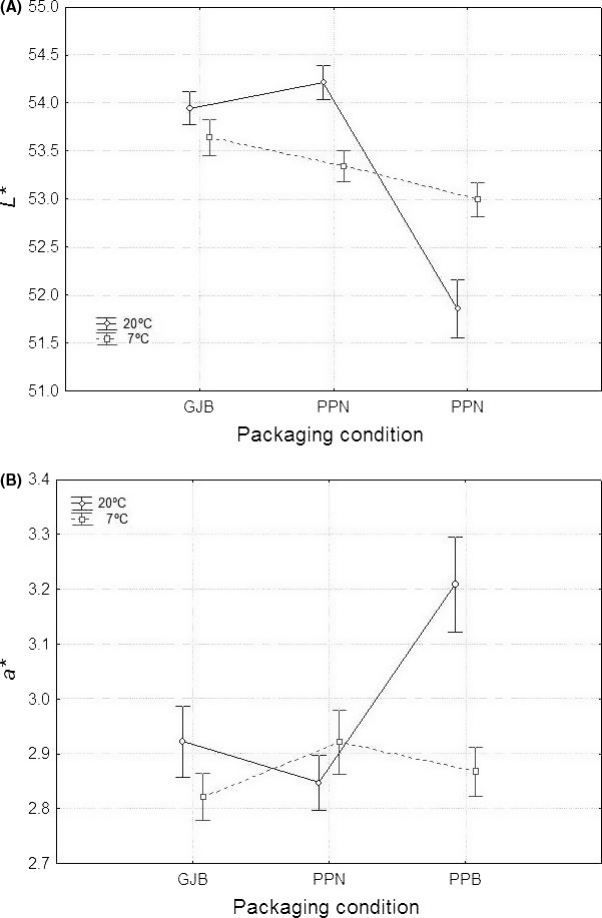
Overall average (*n* = 28) changes in L* (panel A) and a* (panel B) as a function of the packaging conditions, according to temperature. GJB, glass jars with brine; PPB, plastic pouches with brine; PPN, plastic pouches under nitrogen atmosphere; weighted means; vertical bars denote ± standard errors.

**Figure 9:**
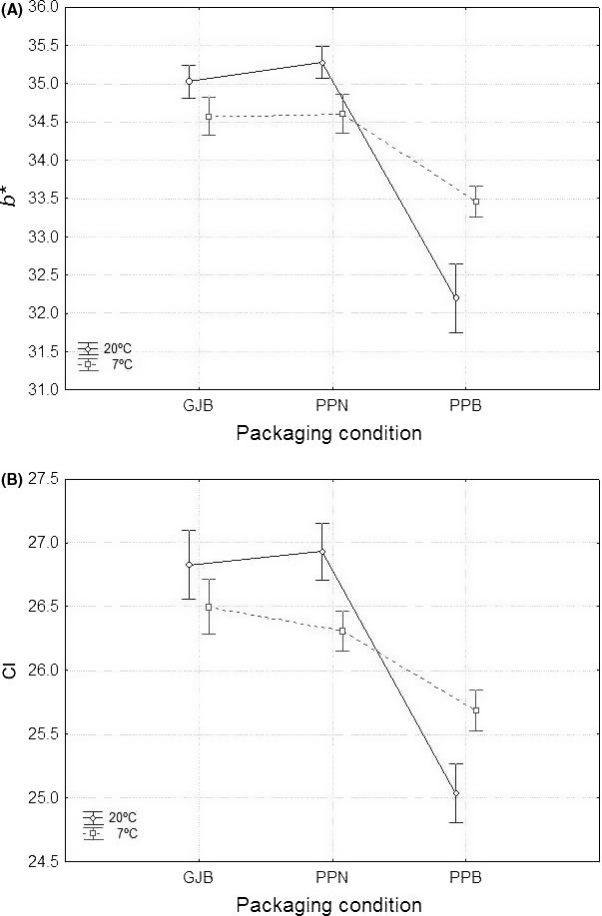
Overall average (*n* = 28) changes in b* (panel A) and Color Index (panel B) as a function of the packaging conditions, according to temperature. GJB, packaging in glass jars with brine; PPB, packaging in plastic pouches with brine; PPN, packaging in plastic pouches under nitrogen atmosphere; weighted means; vertical bars denote ± standard errors.

The analysis of the effect of temperature on color can be finished by displaying the packaging condition*temperature interaction (Figs. 8, 9). In these cases, the effect of time is included in the respective errors. According to both figures, when the packaging material was glass (GJB) none of the color parameters was affected by temperature. In olives packaged under nitrogen atmosphere (PPN), L*, b*, and *CI* have significantly higher values at 20°C (better color) while no effect was noticed on the a* values of these fruits; therefore, olives from PPN had, in general, better color when stored at 20°C. Finally, in olives packaged in PPB, L*, b*, and *CI* had lower values (poorer color) at 20°C while a* showed higher levels (also poorer colour) at this temperature. Thus, when using PPB, storage at 7°C was more favorable (higher L*, b* and *CI* values and lower a* level) owing to the lower oxygen content in the brines of the pouches at this temperature. As a result, GJB and PPN had a good behavior with respect to color preservation which even improved in PPN when stored at 20°C (a temperature which is also convenient for LAB survival, as mention above). On the contrary, packaging in PPB was markedly affected by temperature and preservation or color improvement required refrigeration (storage at 7°C); but this cannot be considered as a good option because of the lower LAB survival and null LAB2 imposition at this temperature. Undoubtedly, this packaging condition is not recommendable for any type of green Spanish‐style table olive where color is the attribute to be preserved.

## Discussion

In table olives, research has demonstrated that the packaging conditions can significantly influence the physicochemical and microbiological characteristics of the final products (Panagou 2004; Doulgeraki et al. 2012; Rodríguez‐Gómez et al. 2015). In the case of olives fermented with potential probiotic strains, packaging requires a compatible stabilization/preservation of the packaged products with the optimization of the LAB survival. A first approach to achieving these objectives could be the use of proper physicochemical conditions (Borbolla y Alcalá and González Pellissó 1972). The method is simple and the characteristics of the packaging brines can be estimated easily by means of mass balance, once the equilibrium conditions have been set (Garrido‐Fernández et al. 1997). However, this strategy was developed for the stabilization of packages (that is, at least, for preventing the growth of live organisms) but there is limited experience in its application to potential probiotic olives (e.g., for preserving the olive biofilm). An exploratory research has disclosed promising results and shown that packaging conditions and storage temperature may play an essential role in this aim (Rodríguez‐Gómez et al. 2015). The objective of this work is to go further into these aspects (longer storage period and presence in the final product of the specific putative probiotic strain (LAB2) used to ferment the olives. Apparently, the products have remained stable (physicochemical characteristics) during a period of 8 months, except for titratable acidity which increased in some treatments. In principle, such an effect cannot be considered as a problem but a convenient circumstance for strengthening the product safety, provided it will not produce a markedly negative impact on LAB survival (as observed in GJB). Furthermore, it may be associated with LAB survival and vitality, in agreement with the objectives of the work, because of the strong association between both phenomena in GJB at 20°C. Color is among the most sensible attributes of green Spanish‐style table olives (Sánchez‐Gómez et al. 2013). In this work, the GJB and PPN were the most reliable options with respect to color owing to their favorable effect on its preservation at room temperature. On the contrary, the results advise against the use of PPB in olive packaging in general because it resulted in an important degradation of the color (all parameters) in the long run, due to its permeability to oxygen.

The most important objective when producing probiotic table olives is the preservation of a high LAB population on fruits from fermentation to intake. A progressive decline of both LAB and yeast populations on olives throughout storage is usual (Rodríguez‐Gómez et al. 2015). Based on previous results, this work has controlled the packaging conditions and temperature in order to minimize this effect. In this way, GJB and PPN were able to retain high putative probiotic LAB2 strain populations at 20°C. On the contrary, refrigeration temperatures produced unassumable LAB decreases. Furthermore, the most important and, possibly, critical conclusion has been derived from the interaction of packaging conditions*storage and the temperature effect on LAB2 presence. At room temperature, LAB2 (used for fermentation) constituted 100% of the LAB populations on the olives from GJB and PPN, while it was completely absent in those from PPB. At refrigeration temperature (7°C), LAB2 was also absent, regardless of treatments. Apparently, low temperature and PPB favored the replacement of LAB2 by wild strains better adapted to such environmental conditions. It is the first time that such a determinant effect of storage temperature and packaging conditions is reported to so radically affect the imposition of a selected strain (LAB2). It is obvious that this effect requires further confirmation but diverse responses to environmental conditions of different strains have already been informed. Blana et al. (2014) reported high colonization (80–90%) of *L. pentosus* B281 and B282 on olive fermentation at 8% NaCl but the second microorganism was unable to establish itself on the olive biofilm. The strong negative effect of storage at 7°C on LAB2 presence in the olive biofilm at the end of storage is remarkable and contrasts with the usual distribution throughout the cold chain of probiotic dairy products; in fact, reports about distribution under nonrefrigerated conditions are the exception (Liu and Tsao 2009). Furthermore, temperature abuse is supposed to spoil the products because of the growth of yeasts (Viljoen et al. 2003). In this way, the response of LAB2 to temperature is rather peculiar. In this work, the eventual storage was extended to 8 months. Lavermicocca et al. (2005) recommended a 3‐month period, with a high viability in LAB population. A longer period (270 days) was observed for probiotic LAB in cheddar cheese (Ganesan et al. 2014) but, in general, the survival in cheese is variable and is usually shorter (Karimi et al. 2011). Therefore, the 8‐month table olive storage is a prolonged period which would be longer than most assigned to many other probiotic products.

At room temperature, the decrease in yeasts on olives was very pronounced, except in PPB (because of the possible growth promoting effect of the presence of oxygen, due to plastic permeability) and represents a serious drawback for the development of products containing simultaneously functional LAB and yeast strains. At refrigeration temperature 7°C, the yeast survival was moderate and might be adequate for storing eventual yeast probiotic olives.

There are also other strategies to improve the survival of probiotics in foods. Charalampopoulus et al. (2003) reported the use of malt, wheat, and barley extracts with this objective. Bevilacqua et al. (2010) and Altieri et al. (2011) suggested the addition of amino‐acids and prebiotics, respectively. However, these compounds are hardly applicable to the case of probiotic table olives, where brine transparency and the absence of strange materials are very influential for consumer acceptance. Possibly, a more convenient strategy in table olives would be the enhancement of LAB survival by selected yeasts but, in light of the sensibility shown by the yeasts habitually present in olives, new less common species or strains should be checked. In other products, this option has been successfully applied. The stability of probiotics in fermented milk was improved by *Williopsis saturnus* var. *saturnus* presence (Liu and Tsao 2009, 2010). Furthermore, the addition of *Saccharomyces cerevisiae* had a favorable effect on *Lactobacillus rhamnosus* viability in fermented milk (Suharja et al. 2014).

## Conclusions

In summary, this work has demonstrated that olives initially fermented with potential probiotic bacteria (LAB2) can be packaged for at least for 8 months without apparent product spoilage and quality preservation. Furthermore, the putative probiotic bacteria, used as inoculum in fermentation, was also present at the end of the commercialization period (100% imposition at the end of storage), provided the proper packaging conditions (GJB and PPN) and storage temperature (20°C) were applied. This behavior, which corroborates other previous works on LAB survival for a shorter period of time, may allow an eventual distribution of the new probiotic LAB2 products in common packaging containers without refrigeration. On the contrary, the eventual use of mixture cultures (LAB and yeasts) for the production of potential probiotic olives is not straightforward due to a possible opposed response to packaging conditions and storage temperatures between both groups of microorganisms.

## Conflict of Interest

The authors declare no conflict of interest.
